# Supplementary material for: Reciprocal inhibition of YAP/TAZ and NF-κB regulates osteoarthritic cartilage degradation
Source: Nat Commun. 2018 Nov 1;9:4564. doi: 10.1038/s41467-018-07022-2 (PMC6212432; doi:10.1038/s41467-018-07022-2)
Supplement: Supplementary file 1 — Supplementary Information [file 41467_2018_7022_MOESM1_ESM.pdf]

SUPPLEMENTARY INFORMATION

Reciprocal inhibition of YAP/TAZ and NFκB regulates osteoarthritic cartilage degradation

Deng et al.

Supplementary Figure 1

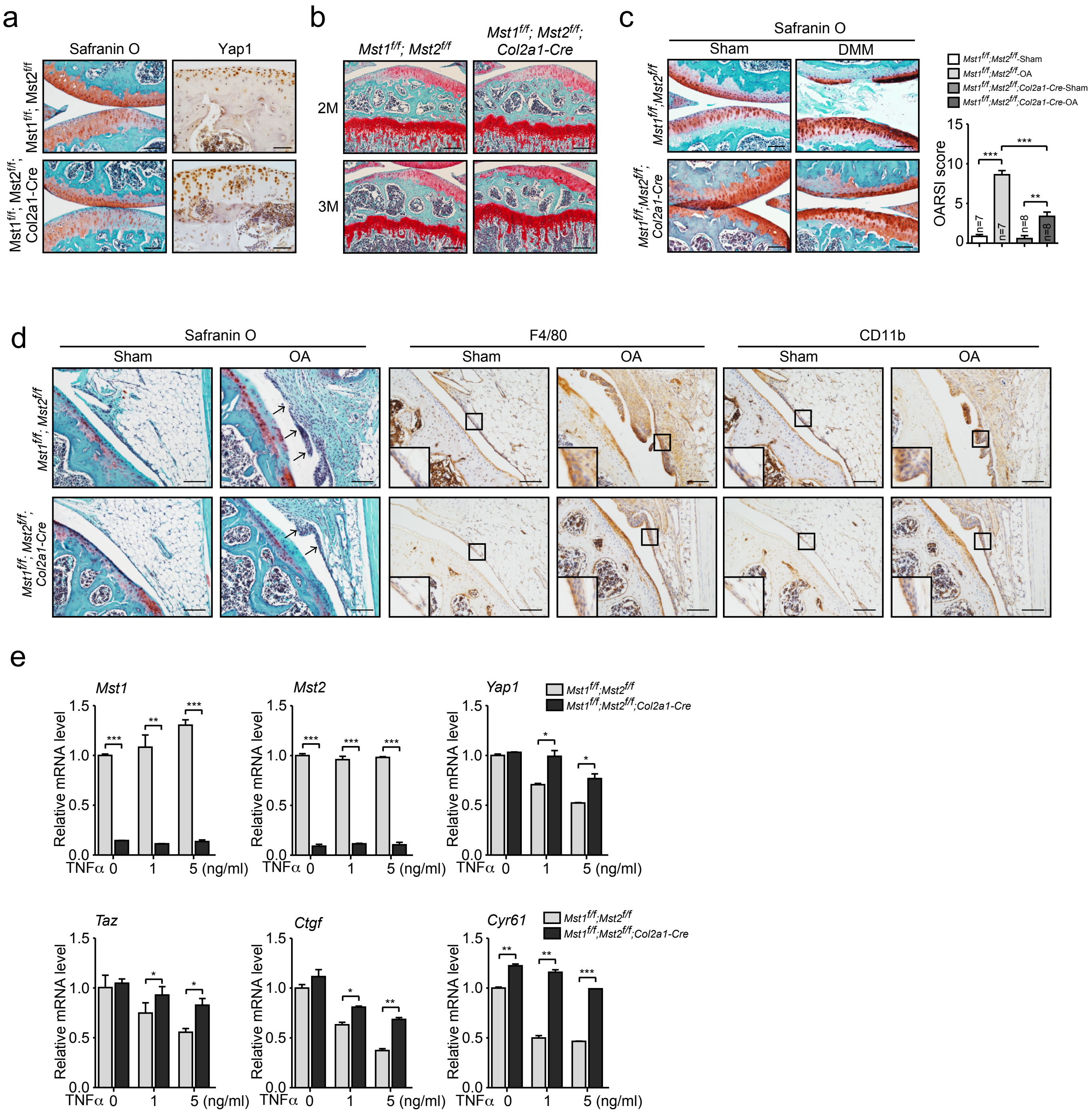

Supplementary Figure 1. Genetic removal of Mst1/2 kinases in chondrocytes attenuates the expression of OA related genes and inhibits synovitis in OA.

(a) Safranin O staining (Left) and immunohistochemistry analysis of YAP (Right) in sagittal sections of the knee joints of the mice with genotypes as shown at 2-month old. Scale bars, 50 μm (n=6 per group). (b) Safranin O staining of the tibia sections of the mice with genotypes as shown at 2 and 3 months old. Scale bars, 100 μm (n=3 per group). (c) Safranin O staining (Left) of sagittal sections of knee joints of the mice with genotypes as shown 10 weeks after DMM surgery. Scale bars, 50μm. OARSI scores (Right) of samples shown in left. (d) Safranin O staining (Left) and immunohistochemistry analysis of macrophage markers F4/80 (Middle) or CD11b (Right) in the synovial membrane of the mice with genotypes as shown 2 months after OA surgery. Inflammation was less severe in the peripheral fibrous tissues with fewer synovial lining cells (black arrows) in the synovial joint of the *Mst1*$^{f/f}$*; Mst2*$^{f/f}$*; Col2a1-Cre* mice with OA. Scale bars, 100μm (n=3 per group). (e) Gene expression analysis of *Mst1/2*, *Yap1/Taz* and *Yap1* target genes in primary chondrocytes after treatment with TNFα for 24 hrs. All results were repeated 3 times and are shown as mean ± SD. *$p<0.05$, **$p<0.01$, ***$p<0.001$. For (c), One-way ANOVA followed by Tukey's test was performed. For (e), two-tailed Student's *t*-test was performed.

Supplementary Figure 2

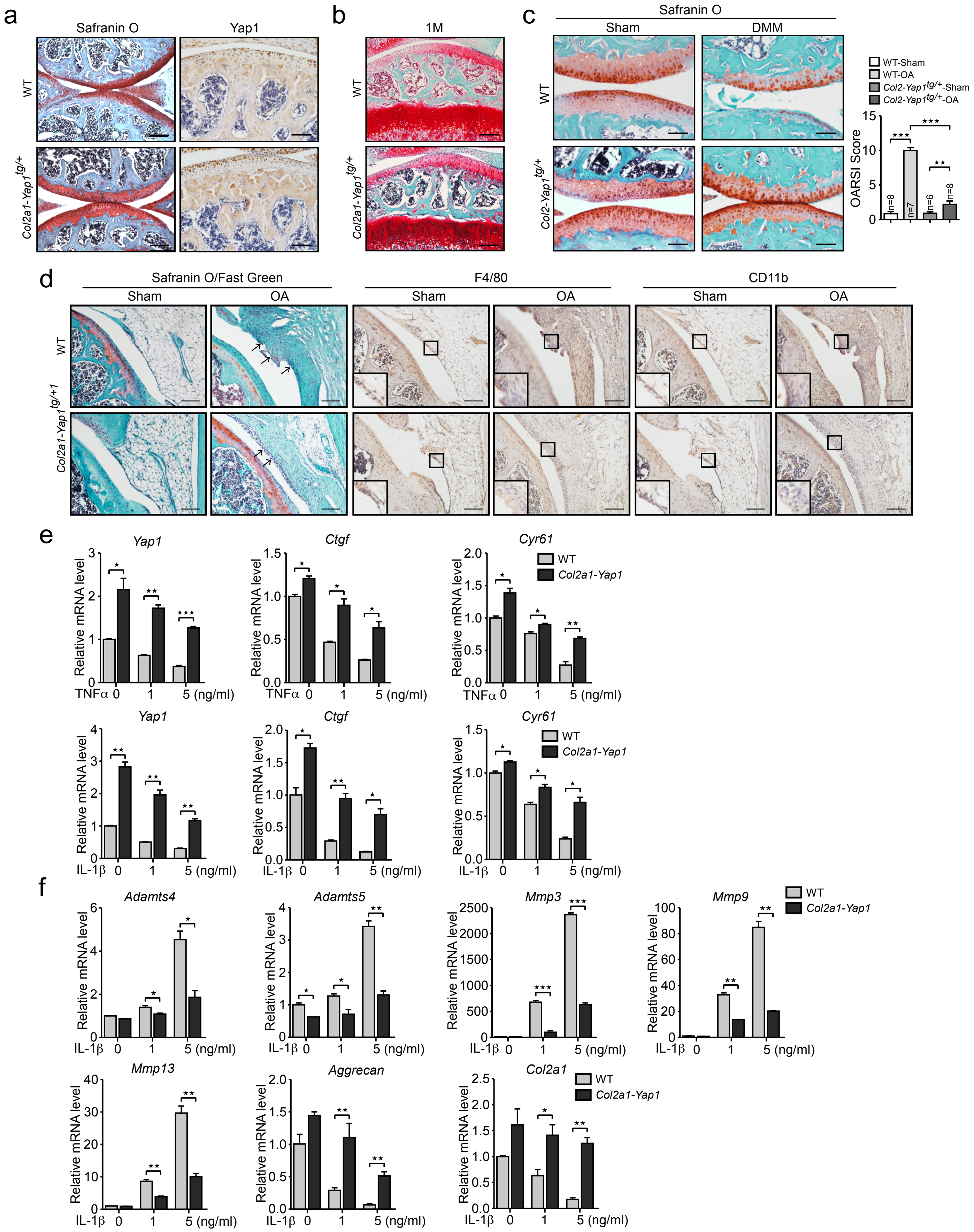

Supplementary Figure 2. YAP overexpression in chondrocytes attenuates the expression of OA related genes and inhibits synovitis in OA.

(a) Safranin O staining (Left) and immunohistochemistry analysis of Yap1 (Right) in sagittal sections of the knee joints of *Col2a1-Yap1* transgenic mice at 2 months old. Scale bars, 100μm (n=6 per group). (b) Safranin O staining of the tibia sections of the mice with genotypes as shown at 1 month old. Scale bars, 100 μm (n=3 per group) (c) Safranin O staining of sagittal sections of knee joints of wild-type and *Col2a1-Yap1*$^{tg/+}$mice 10 weeks after DMM surgery. Scale bars, 100μm. OARSI scores of samples shown in right panel. (d) Safranin O staining (Left) and immunohistochemistry analysis of macrophage marker genes F4/80 (Middle) and CD11b (Right) on the synovial membrane of mice with genotypes as shown 2 months after OA surgery. Inflammation was less severe in the peripheral fibrous tissues with fewer synovial lining cells (black arrows) in the synovial joint of the *Col2a1-Yap1*$^{tg/+}$ mice with OA. Scale bars, 100μm (n=3 per group). (e) Gene expression analysis of *Yap1* and its target genes in primary chondrocytes after treatment with TNFα (A) or IL-1β (B) for 24 hrs as shown. (f) Gene expression analysis of matrix-degrading enzymes and matrix components in primary chondrocytes after treatment with IL-1β for 24 hrs. All results were repeated 3 times and shown as mean ± SD. **p*<0.05, ***p*<0.01, ****p*<0.001. For (c), One-way ANOVA followed by Tukey's test was performed. For (e, f), two-tailed Student's *t*-test was performed.

Supplementary Figure 3

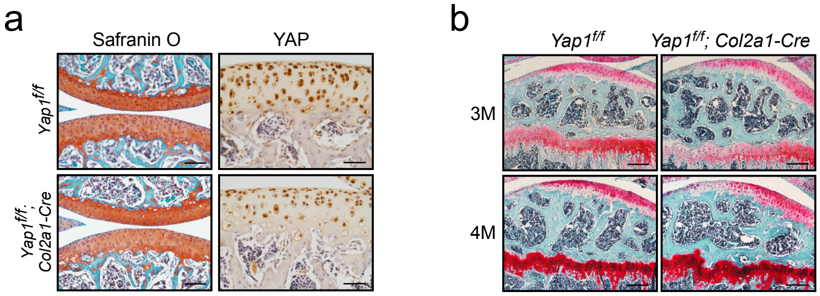

Supplementary Figure 3. Normal articular cartilage formation in adult *Yap$^{f/f}$; Col2a1-Cre* mutant mice.

(a) Safranin O staining (Left) and immunohistochemistry analysis of Yap1 (Right) in sagittal sections of the knee joints of *Yap1$^{f/f}$; Col2a1-Cre* mice at 1 month old. Scale bars, 100μm (Left), 50μm (Right) (n=3 per group). (b) Safranin O staining of the tibia sections of the mice with genotypes as shown at 3 and 4 months old. Scale bars, 100 μm (n=3 per group)

Supplementary Figure 4

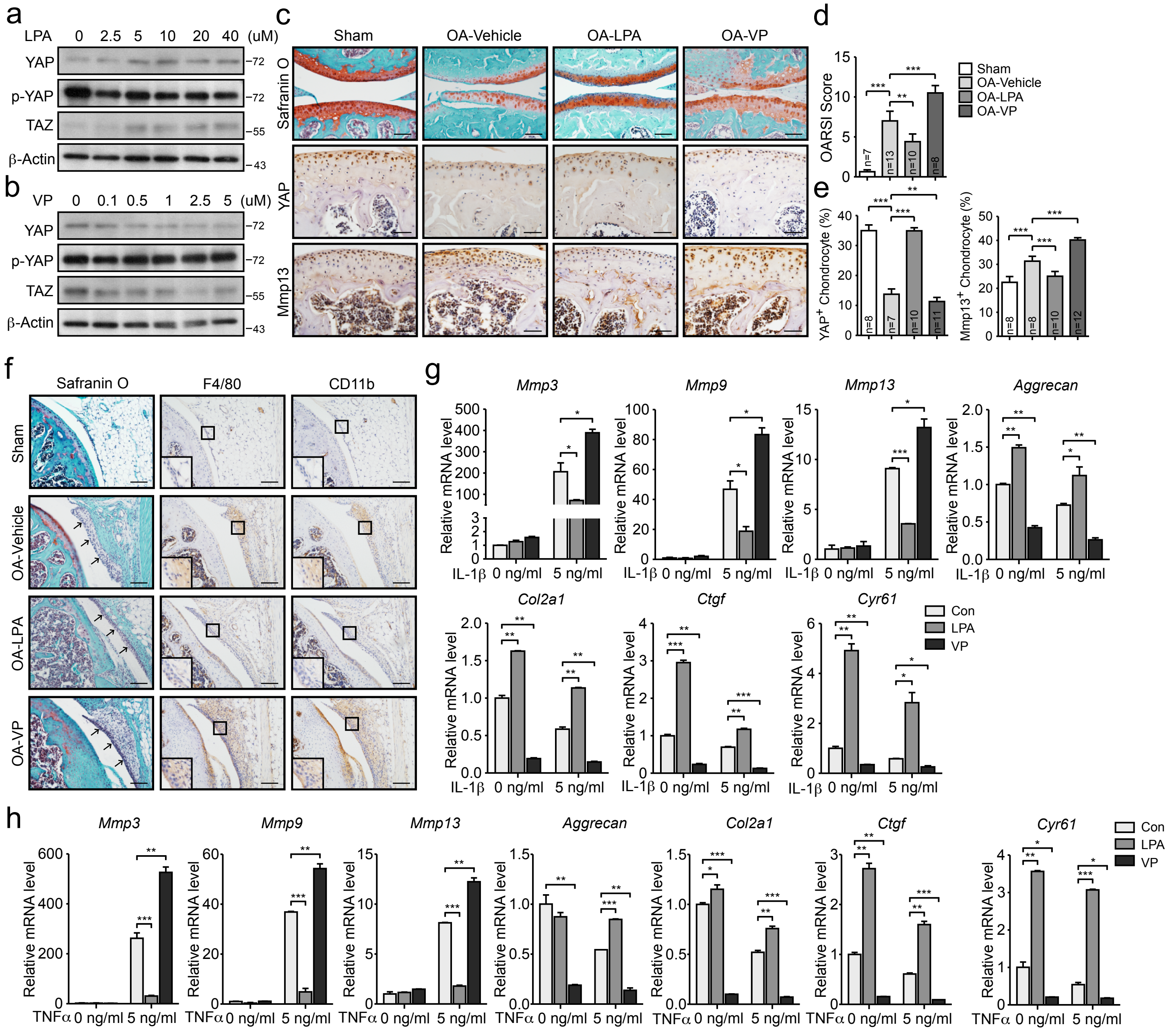

Supplementary Figure 4. Pharmacological regulation of YAP activity controls articular cartilage homeostasis.

(a) Immunoblot analysis of YAP or TAZ expression in primary chondrocytes with serum starvation and treated with LPA for 2 hrs with concentration as shown. (b) Immunoblot analysis of YAP and TAZ expression in primary chondrocytes treated with VP for 6 hrs with concentration as shown. (c) Safranin O staining (Top) and immunohistochemistry of YAP or Mmp13 (Bottom) (brown) in the articular cartilage of the knee joints of wild-type mice with ACLT surgery and treated with alginate beads loaded with Vehicle, LPA (25 μg/mice) or VP (5 μg/mice) respectively. Scale bars, 100 μm (Top) and 50 μm (Bottom).

(d and e) OARSI scores (d) and statistical analysis of the percentage of YAP^+ or $Mmp13^+$ chondrocytes (e) of samples shown in (c). (f) Safranin O staining (Left) and immunohistochemistry analysis of macrophage markes F4/80 (Middle) or CD11b (Right) of the synovial membrane of wild-type mice 2 months after OA surgery with treatment of Vehicle, LPA or VP respectively. Inflammation was less severe in the peripheral fibrous tissues with fewer synovial lining cells (black arrows) in the synovial joint of the LPA-treated mice with OA. Scale bars, 100μm (n=3 per group). (g and h) Gene expression of matrix degrading enzymes, extracellular matrix components and YAP target genes in articular chondrocytes pre-treated with LPA (20 μM) or VP (1 μM) for 2 hrs respectively, followed by IL-1β (G) and TNFα (H) treatment for 6 hrs. All results were repeated 3 times and shown as mean ± SD. *p<0.05, **p<0.01, ***p<0.001. For (d and e) One-way ANOVA followed by Tukey's test was performed. For (g and h), One-way ANOVA followed by Dunnett's test was performed.

Supplementary Figure 5

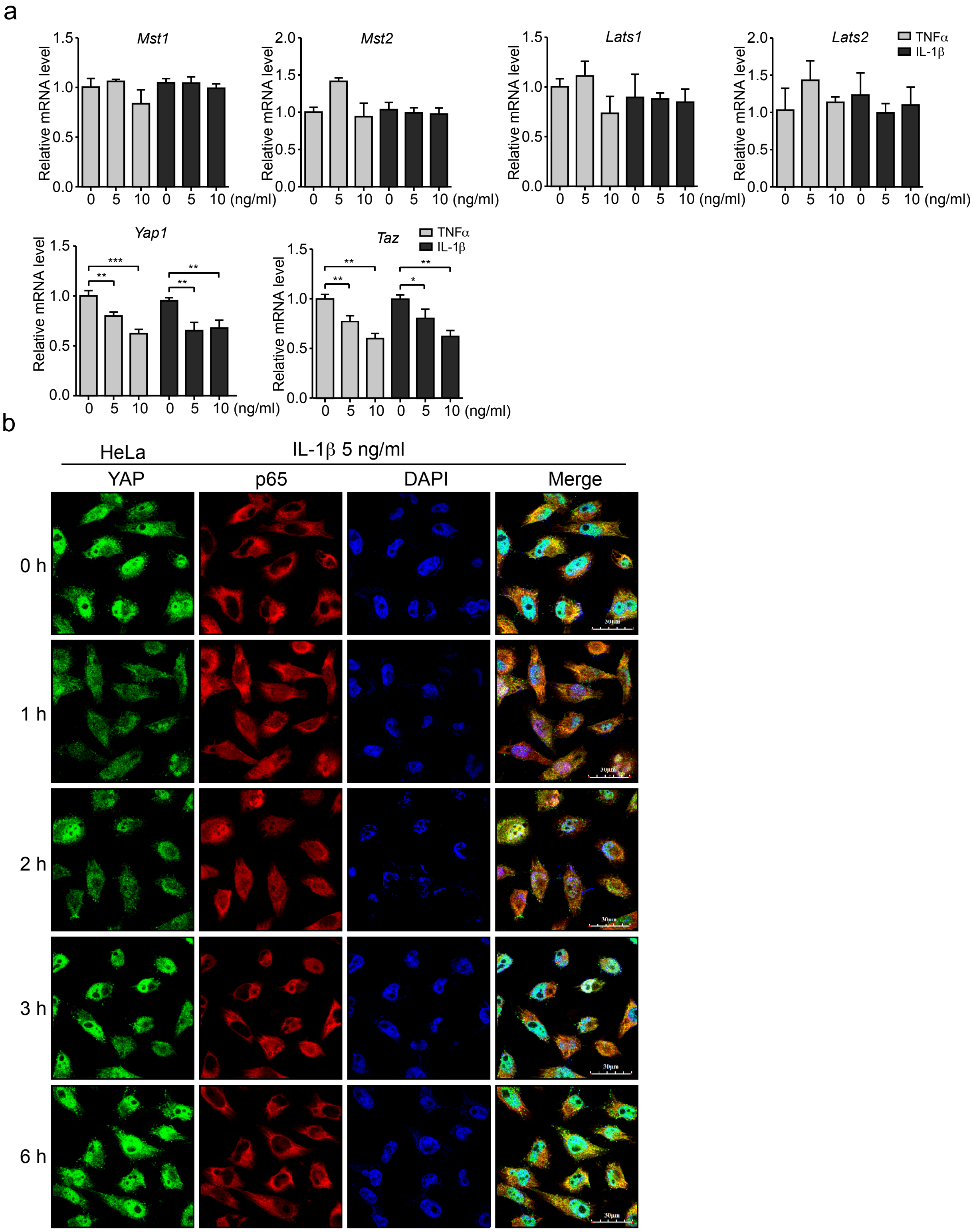

Supplementary Figure 5. Inflammatory cytokines suppress the expression of *Yap1*/*Taz* in articular chondrocytes.

(a) Gene expression analysis of primary chondrocytes treated with TNFα or IL-1β at indicated concentration for 24 hrs. (b) Immunofluorescence staining of YAP and p65 in HeLa cells treated with IL-1β at 5 ng/ml for indicated time. Scale bars, 30 μm. All results were repeated for 3 times and shown as mean ± SD. *p<0.05, **p<0.01, ***p<0.001, For (a), One-way ANOVA followed by Dunnett's test was performed.

Supplementary Figure 6

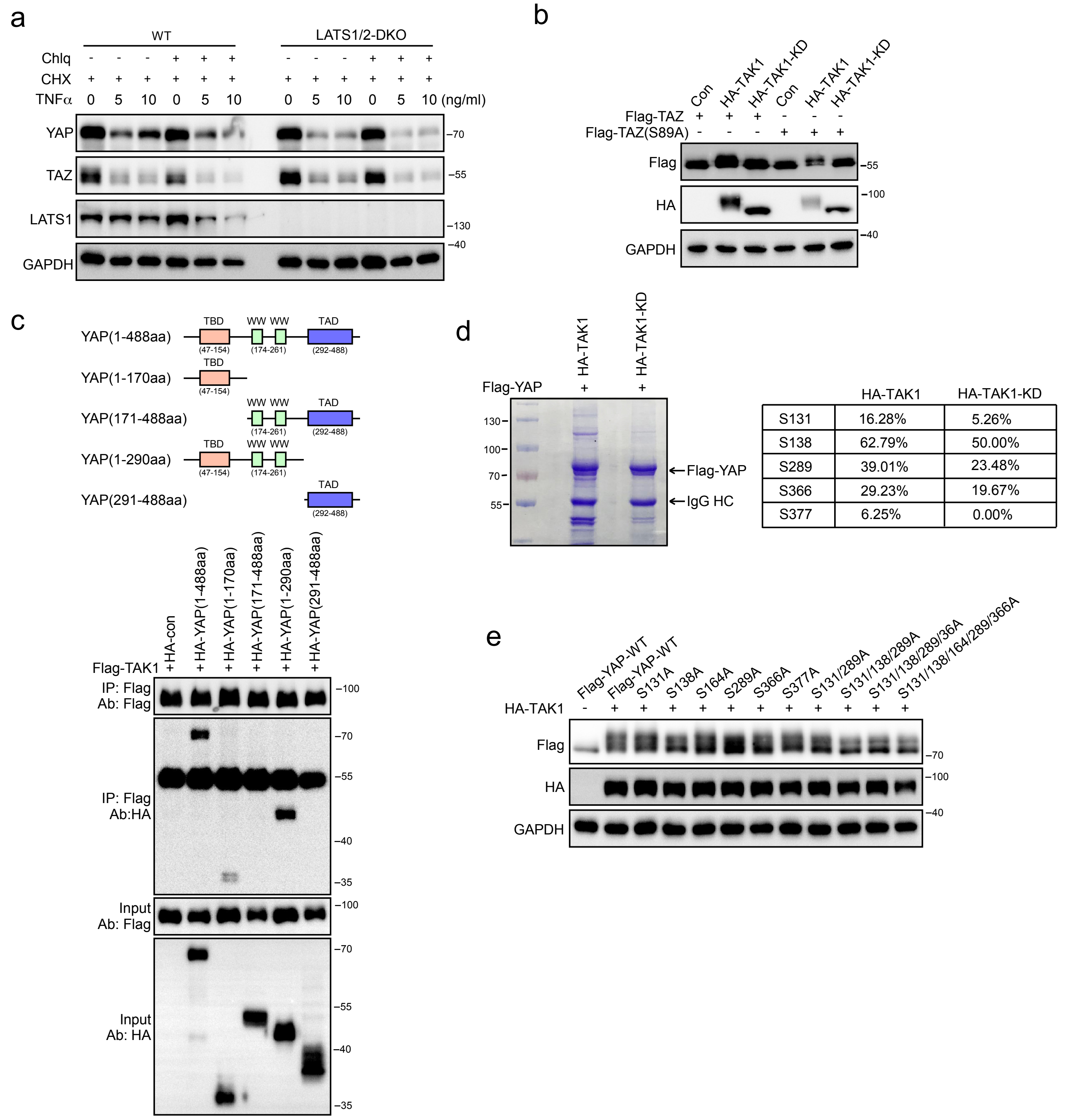

	HA-TAK1	HA-TAK1-KD
S131	16.28%	5.26%
S138	62.79%	50.00%
S289	39.01%	23.48%
S366	29.23%	19.67%
S377	6.25%	0.00%

Supplementary Figure 6. TAK1 phosphorylates YAP/TAZ independent of LATS1/2.

(a) Western blot analysis of YAP and TAZ in WT and LATS1/2-DKO HEK293A cells treated with CHX and Chlq (chloroquine) for 2 hrs followed by treatment with TNFα for 6 hrs. (b) Western blot analysis of lysate of HEK293T transfected with indicated plasmids. (c) Serial truncations of YAP were generated and subjected to immunoprecipitation in HEK293T co-transfected with Flag-tagged TAK1. (d) Immunoprecipitation assay of Flag-tagged YAP with wild-type or kinase-dead TAK1 in HEK293T cells and subjected to SDS-PAGE. Coomassie brilliant blue staining (Top) and Nano-Liquid Chromatography/Mass Spectrometry analysis of enhanced phosphorylation sites in YAP protein (Bottom). (e) Western blot analysis of lysate of HEK293T cells transfected with TAK1 and wild-type or mutant YAP. All results were repeated for 3 times.

Supplementary Figure 7

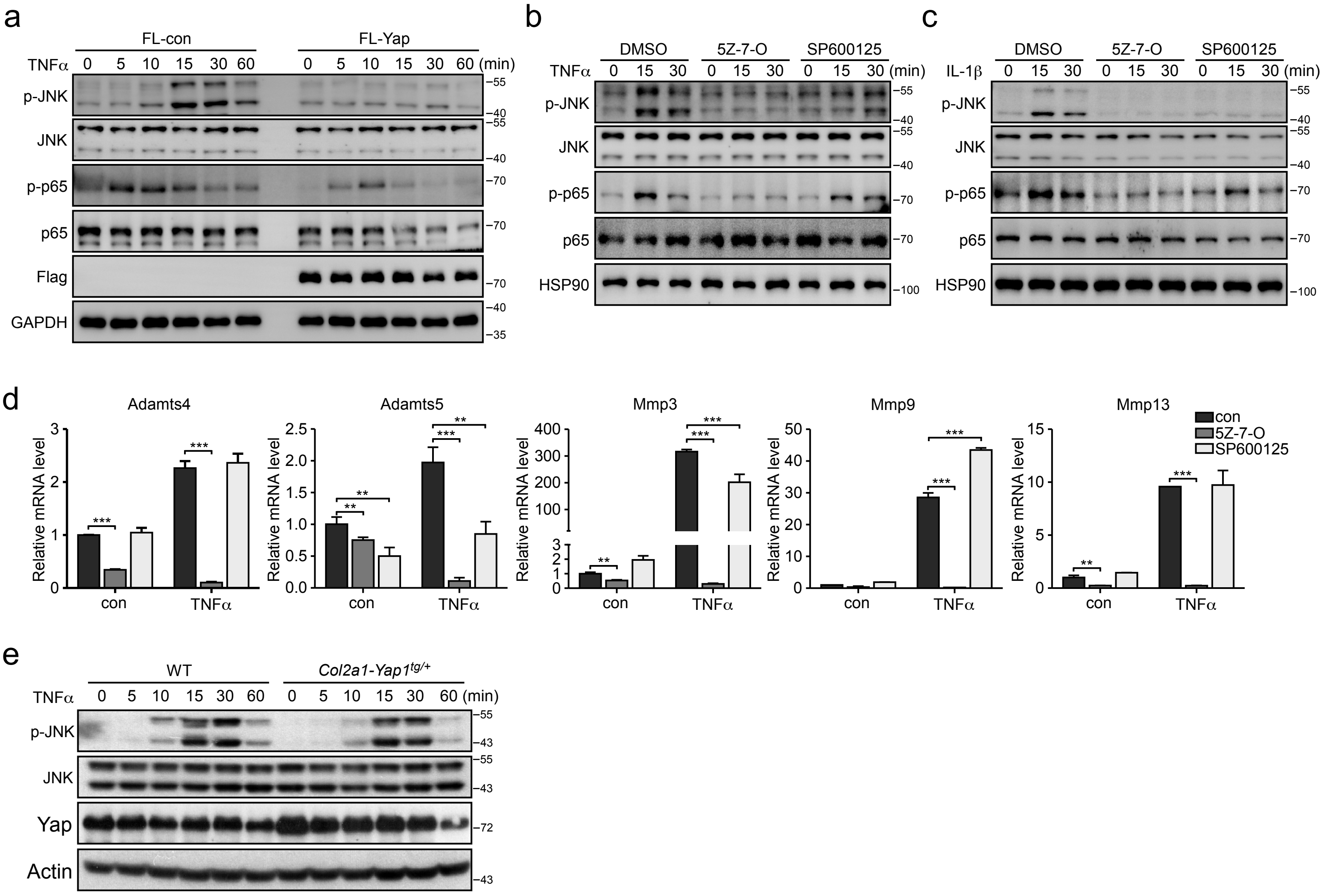

Supplementary Figure 7. YAP inhibits TNFα-induced signaling activation.

(a) Immunoblot analysis of JNK and NF-κB activation in primary chondrocytes transfected with Flag-tagged YAP followed by treatment with TNFα with indicated time as shown. (b-c) Western blot analysis of phosphorylated JNK and p65 in primary chondrocytes treated with TAK1 inhibitor 5Z-7-O at 1 μM or JNK inhibitor SP600125 at 10 μM for 30 mins respectively and then treated with TNFα (b) or IL-1β (c) at 5 ng/ml with indicated time as shown. (d). Gene expression analysis of matrix-degrading enzymes in primary chondrocytes pretreated with TAK1 inhibitor 5Z-7-O at 1 μM or JNK inhibitor SP600125 at 10 μM for 30 mins and followed by treatment with TNFα at 5 ng/ml for 24 hrs. All experiments were repeated three times independently and presented as mean ± SD. *$p<0.05$, **$p<0.01$, ***$p<0.001$. For (d), One-way ANOVA followed by Dunnett's test was performed

Supplementary Figure 8

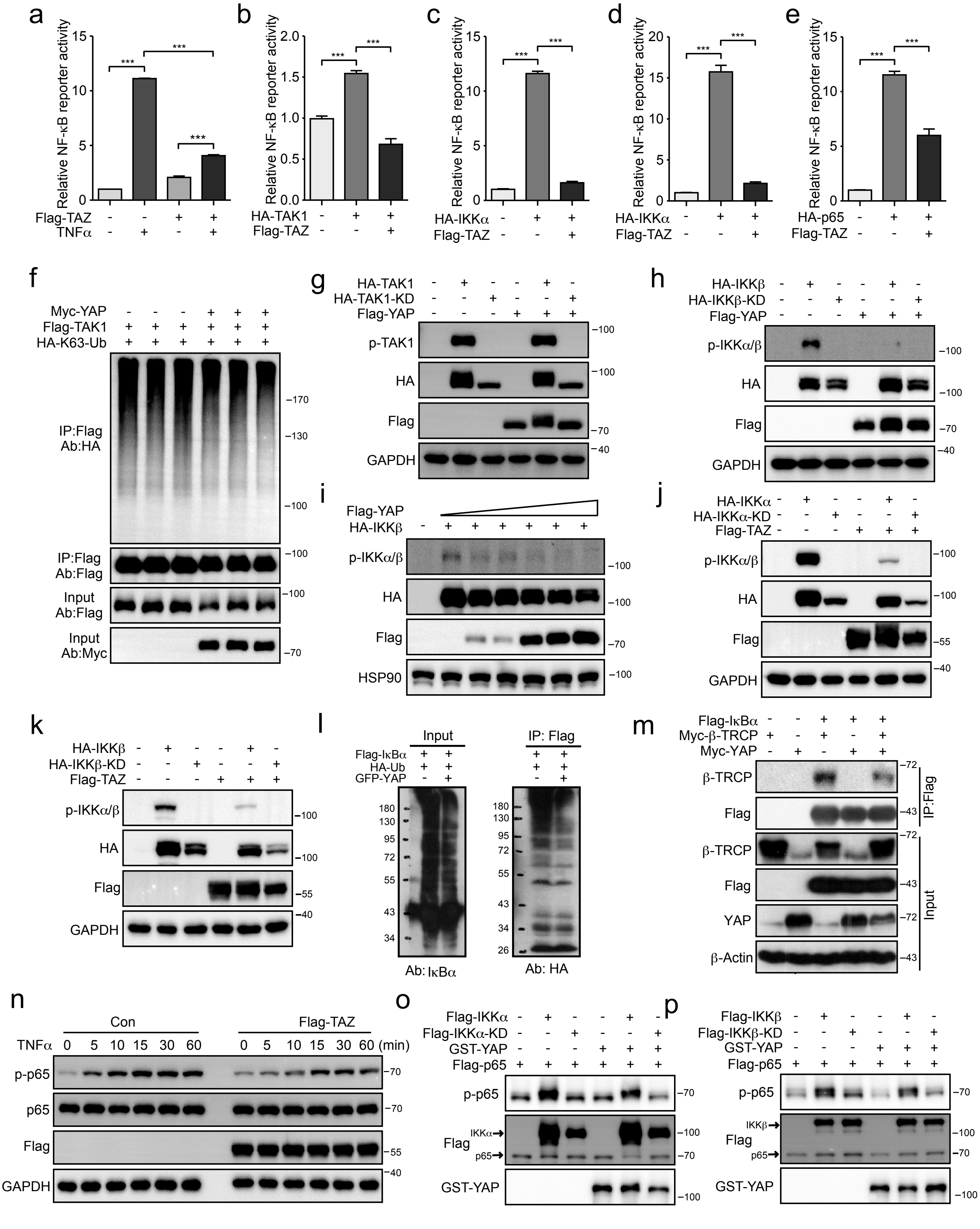

Supplementary Figure 8. YAP/TAZ inhibits NF-κB signaling activity.

(a) Luciferase assay of NF-κB luciferase reporter in HEK293A cells transfected with Flag-tagged TAZ plasmid followed by treatment with TNFα for 6 hrs before collecting cell lysates. (b-e) Luciferase assay of NF-κB luciferase reporter 24 hrs after transfection of TAZ with respective NF-κB component plasmids as indicated in HEK293T cells. (f) K63-ubiquitination assay of TAK1 in HEK293T cells transfected with HA-tagged K63-Ub and pretreated with MG132 for 6 hrs before collection. (g) Western blot analysis of phosphorylated TAK1 in HEK293A cells transfected with or without YAP. (h) Immunoblot analysis of the phosphorylation of IKKβ with overexpression of YAP in HEK293T cells. (i) Immunoblot analysis of the phosphorylation of IKKβ with expression of different dose of YAP in HEK293T cells. (j and k) Western blot analysis of phosphorylated IKKα (j) and IKKβ (k) in HEK293A cells transfected with or without TAZ. (l) Ubiquitin assay of IκBα with YAP overexpression in primary chondrocytes after treatment with MG132 (10 μM) for 6 hrs and then treated with TNFα at 5 ng/ml for 30 mins. (m) Immunoprecipitation assay of IκBα and β-TRCP with or without YAP expression in HEK293T cells. (n) Western blot analysis of phosphorylated p65 in HEK293A cells transfected with or without TAZ for 24 hrs and then treated with TNFα for different time point as indicated. (o and p) YAP inhibits p65 phosphorylation by IKKα and IKKβ. *In vitro* kinase assay of IKKα (o) and IKKβ (p) using Flag-tagged p65 as substrate with or without recombinant GST-YAP. All results were repeated for 3 times and shown as mean ± SD. *$p<0.05$, **$p<0.01$, ***$p<0.001$. For (a-e) One-way ANOVA followed by Tukey's test was performed.

Supplementary Figure 9

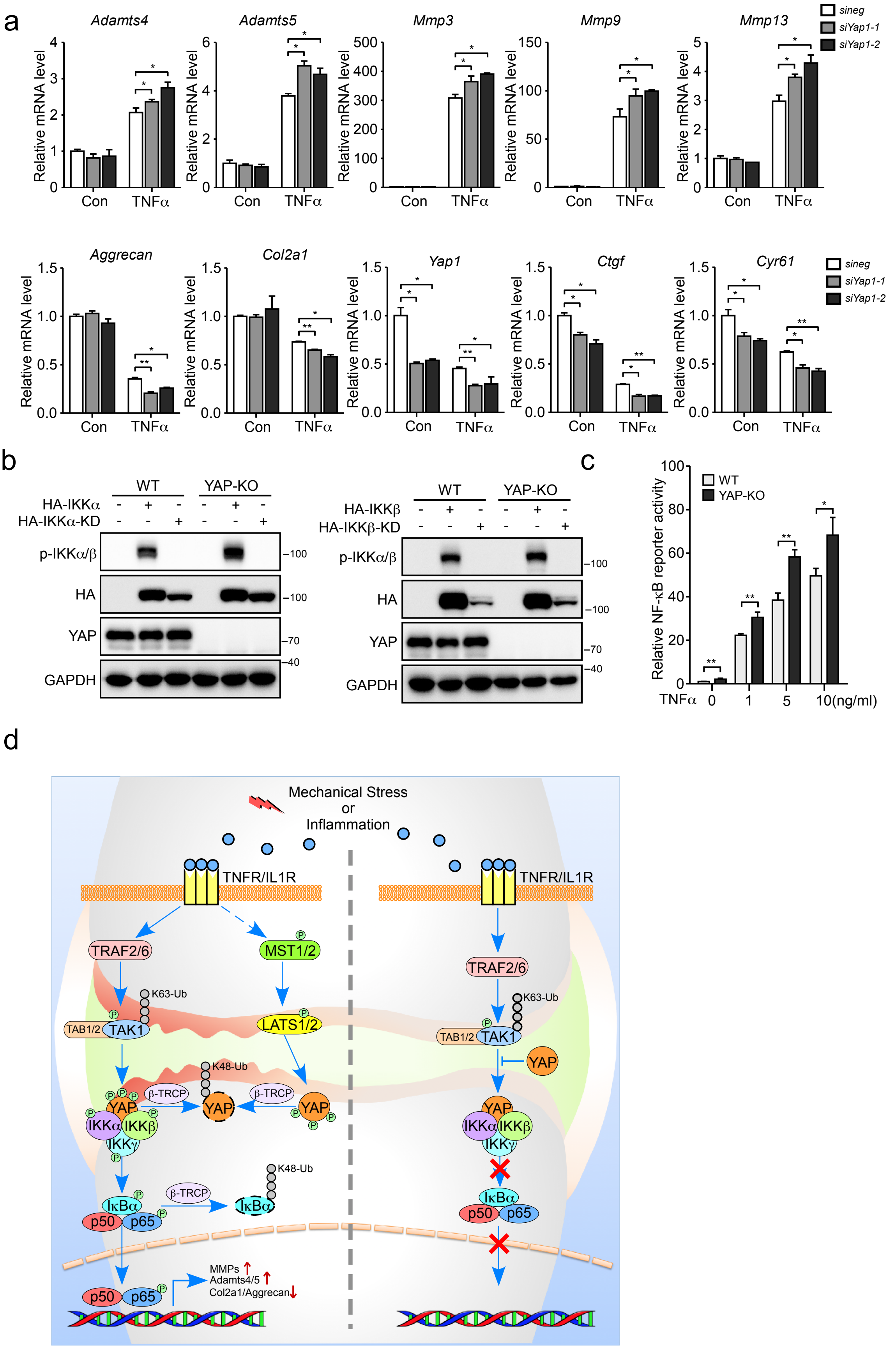

Supplementary Figure 9. Loss of YAP enhances NF-κB signaling activity.

(a) Gene expression analysis of matrix-degrading enzymes, extracellular matrix components and YAP target genes in primary chondrocytes transfected with YAP siRNA for 24 hrs and followed by treatment with TNFα for 24 hrs as shown. (b) Western blot analysis of phosphorylated IKKα and IKKβ in wild-type and YAP-KO HEK293T cells. (c) Luciferase assay of NF-κB reporter activity after treatment with TNFα at indicated concentration for 6 hrs in WT or YAP-KO HEK293T cells. (d) A model of reciprocal antagonism between Hippo-YAP/TAZ and NF-κB pathway. Upon mechanical stress or inflammatory stimuli, immune cells secrete cytokines and trigger inflammatory response. YAP associates with TAK1 to prevent IKKα/β activation and subsequent NF-κB translocation into the nucleus. As a result, the expression of matrix degrading enzymes is inhibited. Reciprocally, TAK1 directly phosphorylates YAP and promotes proteasomal degradation of YAP. All experiments were repeated three times independently and presented as mean ± SD. *$p<0.05$, **$p<0.01$, ***$p<0.001$. For (a), One-way ANOVA followed by Dunnett's test was performed. For (c), two-tailed Student's *t*-test was performed.

Supplementary Figure 10 Uncropped blot images of the indicated Figures (page 1of 4)

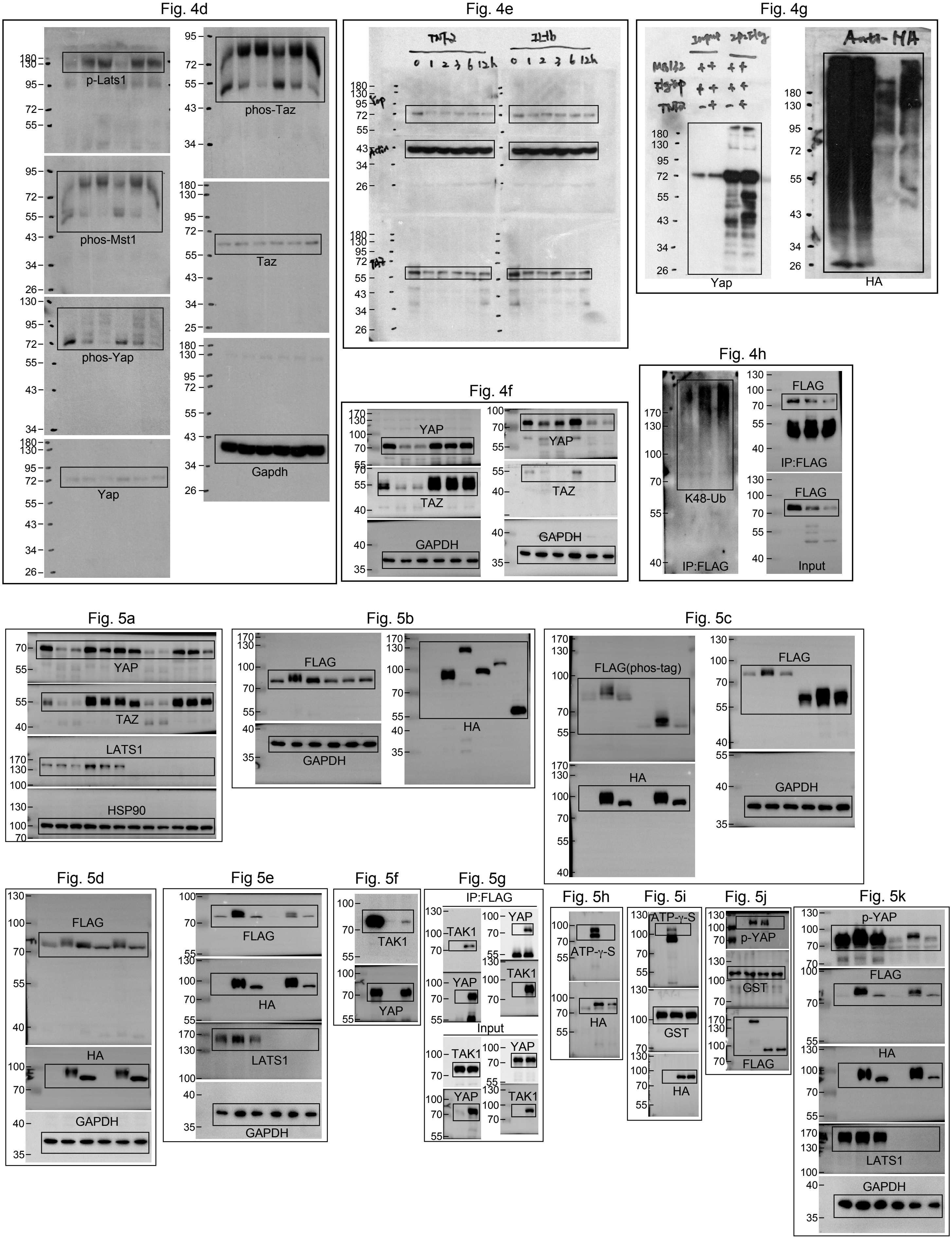

Supplementary Figure 10 Uncropped blot images of the indicated Figures (page 2 of 4)

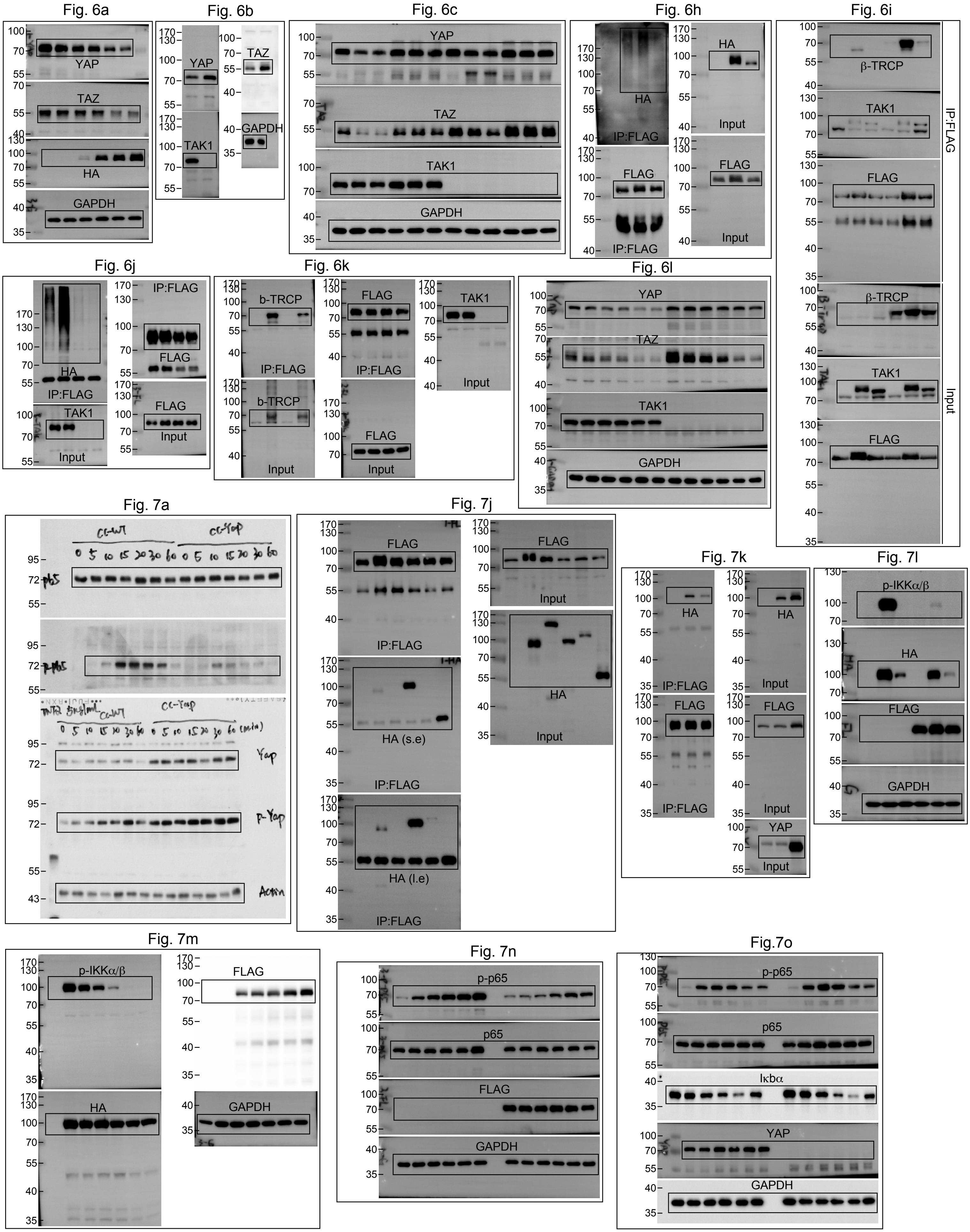

Supplementary Figure 10 Uncropped blot images of the indicated Figures (page 3 of 4)

Supplementary Figure 4a

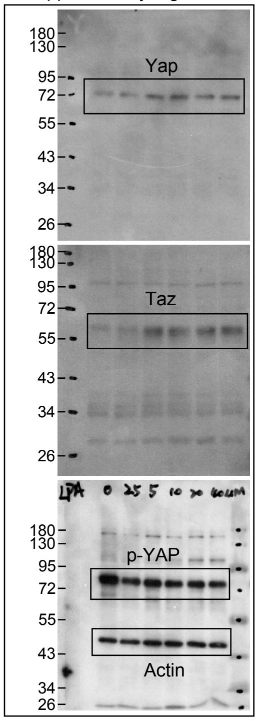

Supplementary Figure 4b

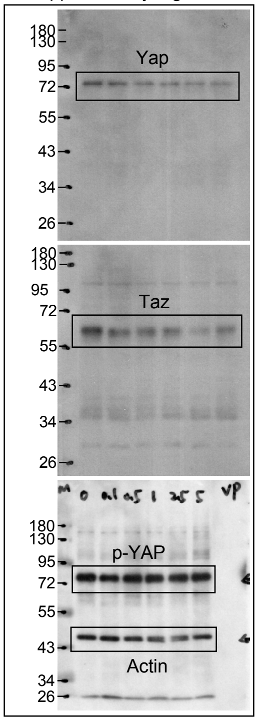

Supplementary Figure 6a

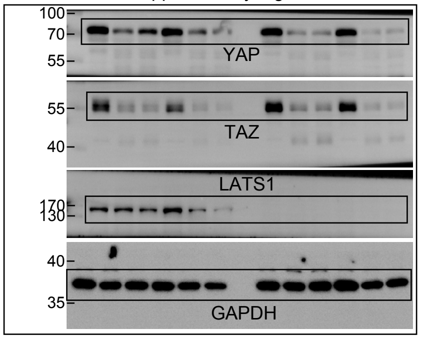

Supplementary Figure 6b

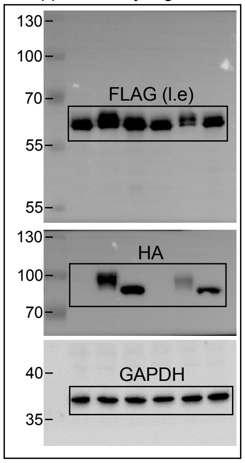

Supplementary Figure 6c

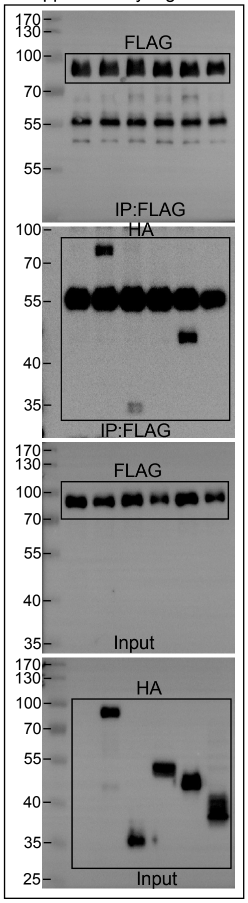

Supplementary Figure 6e

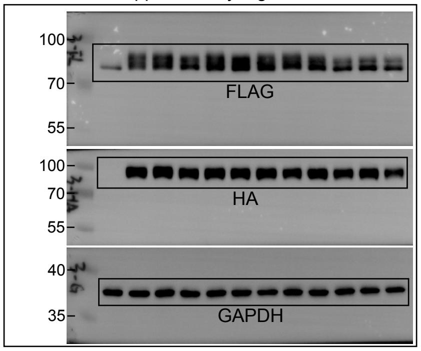

Supplementary Figure 7a

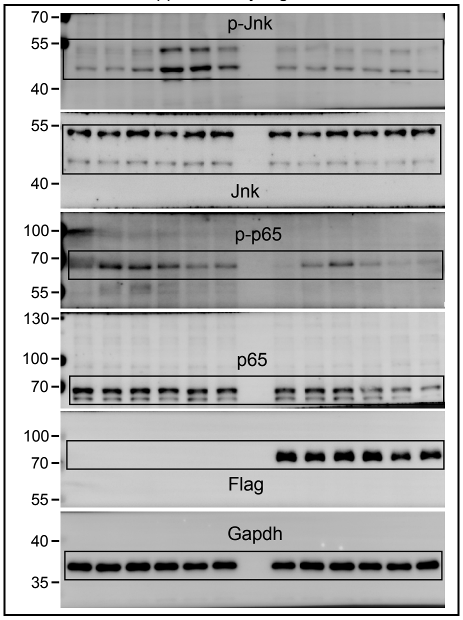

Supplementary Figure 7b

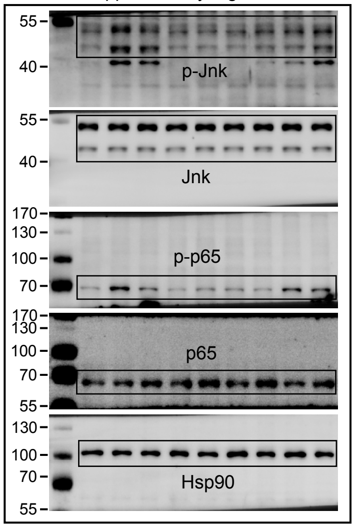

Supplementary Figure 7c

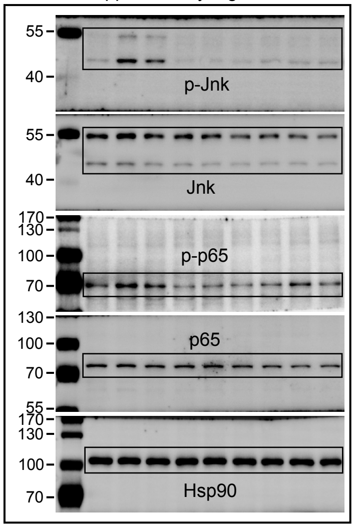

Supplementary Figure 7e

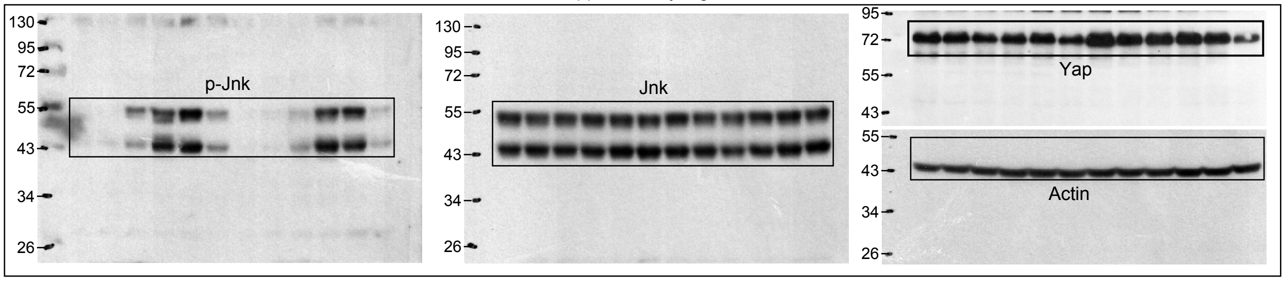

Supplementary Figure 10 Uncropped blot images of the indicated Figures (page 4 of 4)

Supplementary Figure 8f

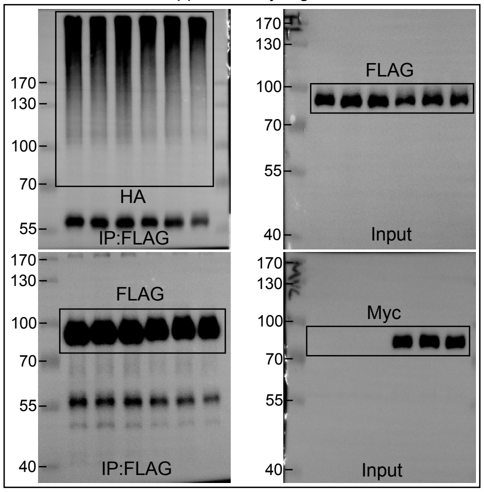

Supplementary Figure 8g

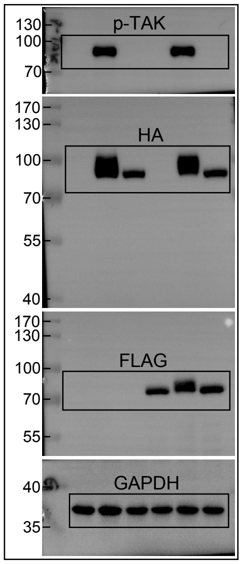

Supplementary Figure 8h

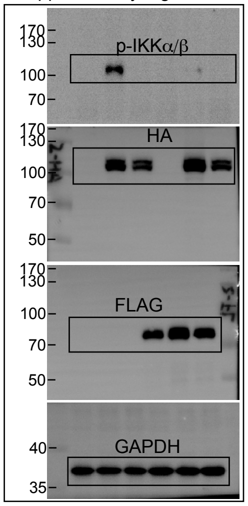

Supplementary Figure 8i

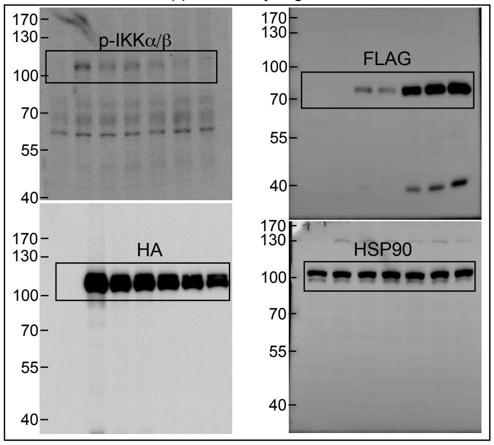

Supplementary Figure 8j

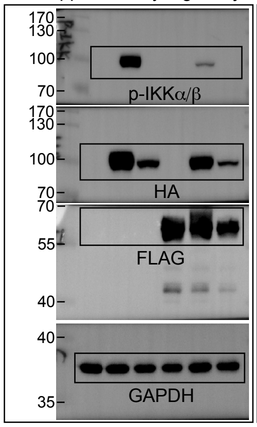

Supplementary Figure 8k

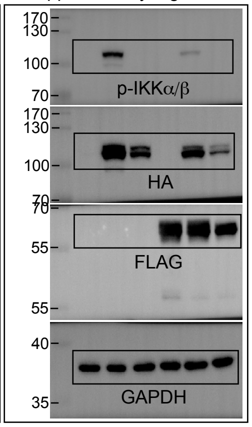

Supplementary Figure 8l

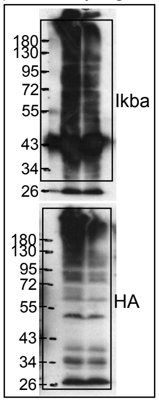

Supplementary Figure 8m

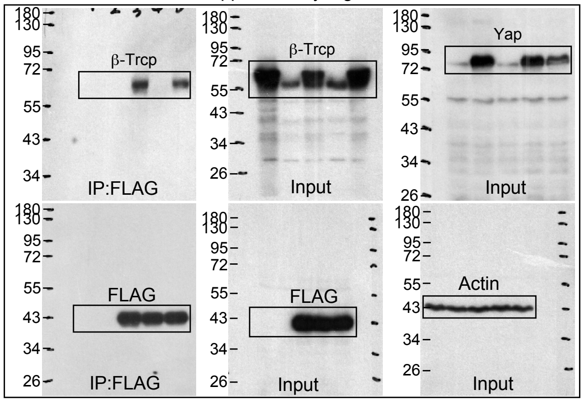

Supplementary Figure 8n

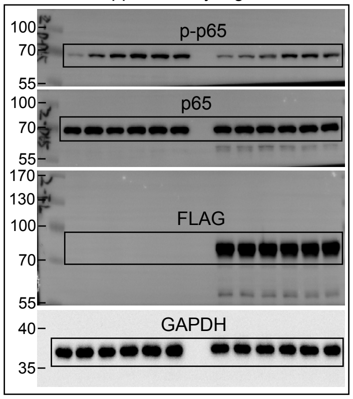

Supplementary Figure 8o

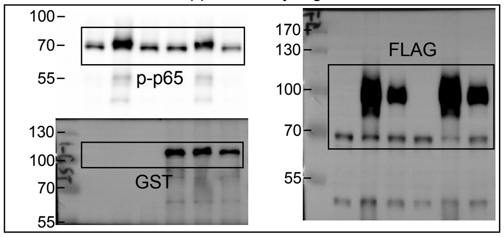

Fig. S8p

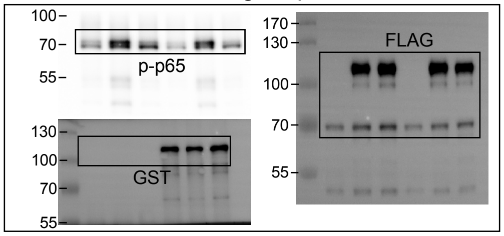

Supplementary Figure 9b (right panel)

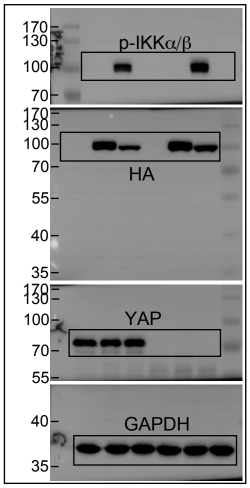

Supplementary Figure 9b (left panel)

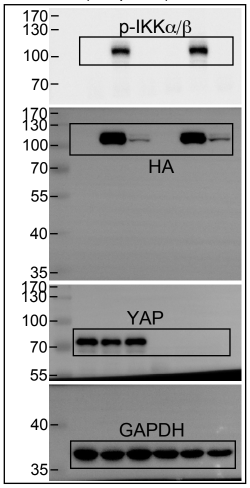

Supplementary Figure 10. Uncropped western blot images of respective figures.

Supplementary Tables

Supplementary Table 1 Q-PCR primer, siRNA and sgRNA target sequence

Q-PCR primer sequence	
**Name**	**Sequence (5'-3')**
Col2a1-F	GACTGAAGGGACACCGAG
Col2a1-R	CCAGGGATTCCATTAGAG
Yap-F	GCTGCAGCAGTTACAGATGG
Yap-R	TGCTCCAGTGTAGGCAACTG
Ctgf-F	GCTGACCTGGAGGAAAAC
Ctgf-R	ACACCCCGCAGAACTTAG
Cyr61-F	CGTCACCCTTCTCCACTT
Cyr61-R	CGTCACCCTTCTCCACTT
Taz-F	GTCCATCACTTCCACCTC
Taz-R	TTGACGCATCCTAATCCT
Mmp3-F	ACTTTGTCCCTTTTGATGG
Mmp3-R	GCCTTGGCTGAGTGGTAG
Mmp9-F	TGTCTGGAGATTCGACTTGAAGTC
Mmp9-R	TGAGTTCCAGGGCACACCA
Mmp13-F	TATGATGGCACTGCTGAC
Mmp-13-R	GTGTAGGTATAGATGGGAAAC
Adamts4-F	CATCCGAAACCCTGTCAACTTG
Adamts4-R	GCCCATCATCTTCCACAATAGC
Adamts5-F	GCCATTGTAATAACCCTGCACC
Adamts5-R	TCAGTCCCATCCGTAACCTTTG
Aggrecan-F	TCCCCAAATCCCTCATAC
Aggrecan-R	TAGTCCACCCCTCCTCAC
Mst1-F	ATTCGGCTACGGAACAAG
Mst1-R	AAAACGGGGTCCCTATTA
Mst2-F	CCCACCACCAACATTCAG
Mst2-R	CATTGTGCCCACGCTTTC
Lats1-F	TCAGTAACGAATGGGGAGA
Lats1-R	GATAATGAGGTCAGAGGCT
Lats2-F	ACCAGAAAGGGAACCACAT
Lats2-R	CCCAACCAGCATCTCAAA
Gapdh-F	TCACTGCCACCCAGAAGAC
Gapdh-R	TGTAGGCCATGAGGTCCAC
hCTGF-F	GTTTGGCCCAGACCCAACTA
hCTGF-R	CTTCTTCATGACCTCGCCGT
hCYR61-F	AGTGGGTCTGTGACGAGGAT
hCYR61-R	GGGTTTCTTTCACAAGGCGG

hGAPDH-F	TGAAGACGGGCGGAGAGAAA
hGAPDH-R	TTCCCGTTCTCAGCCTTGAC
**siRNA sequence**	
**Name**	**Sequence (5'-3')**
siYap-1(sense)	GGCAAUACGGAAUAUCAAU TT
siYap-1(anti-sense)	AUUGAUAUUCCGUAUUGCC TT
siYap-2(sense)	GCGCUGAGUUCCGAAAUCU TT
siYap-2(anti-sense)	AGAUUUCGGAACUCAGCGC TT
**sgRNA target sequence**	
**Name**	**Sequence (5'-3')**
sg-TAK1-1	GAGTTGTTTGCAAAGCTAAG
sg-TAK1-2	AGAGCCTGATGACTCGTTGT
sg-YAP-1	CCAAGGCTTGACCCTCGTTT
Sg-YAP-2	TGGGGGCTGTGACGTTCATC
sg-LATS1	CGTGCAGCTCTCCGCTCTAA
sg-LATS2	TACGCTGGCACCGTAGCCCT

Supplementary Table 2 Antibody information

Name	Source	# Catalog	Dilution
Rabbit Anti-YAP	Novus Biologicals	NB110-58358	IHC (1:100)
Rabbit Anti-MMP13	Abcam	ab39012	IHC (1:100)
Rabbit Anti-F4/80	eBioscience	14-4801-81	IHC (1:100)
Rabbit Anti-CD11b	eBioscience	14-0112-81	IHC (1:100)
Rabbit Anti-YAP	Cell Signaling	4912s	WB (1:1000)
Mouse Anti-YAP	Santa Cruz	sc-101199	WB(1:1000)<br>IF(1:200)
Rabbit Anti- Phospho-YAP	Cell Signaling	4911	WB (1:1000)
Rabbit Anti-TAZ	BD Pharmingen	560235	WB (1:1000)
Rabbit Anti-TAZ	Proteintech	23306-1-AP	WB (1:1000)
Rabbit Anti-MST1	Cell Signaling	3682s	WB (1:1000)
Rabbit Anti-LATS1	Cell Signaling	3477s	WB (1:1000)
Rabbit Anti- Phospho-LATS1	Cell Signaling	8654s	WB (1:1000)
Mouse Anti-NF-κB p65	Cell Signaling	6956s	WB (1:2000)<br>IF (1:200)
Rabbit Anti-NF-κB p65	Cell Signaling	8242s	WB (1:2000)<br>IF (1:200)
Rabbit Anti- Phospho-NF-κB p65	Cell Signaling	3033s	WB (1:2000)<br>IHC(1:100)
Mouse Anti-IkBa	Cell Signaling	4814s	WB (1:2000)
Rabbit Anti-TAK1	Abcam	ab109526	WB (1:1000)
Rabbit Anti- Phospho-TAK1	Cell Signaling	4508s	WB (1:1000)
Rabbit Anti- Phospho-IKKα/β	Cell Signaling	2694s	WB (1:1000)
Rabbit Anti-β-TRCP	Cell Signaling	4394s	WB (1:1000)
Rabbit Anti-β-Actin	Cell Signaling	8457s	WB (1:10000)
Rabbit Anti-GAPDH	Abcam	ab181602	WB (1:10000)
Rat Anti-HA	Roche	11867431001	WB (1:1000)
Rabbit Anti-HA	Proteintech	51064-2-AP	WB (1:1000)
Rabbit Anti-K48-Ub	Cell Signaling	4289s	WB (1:1000)
Mouse Anti-FLAG	Sigma	F3165	WB (1:2000)
Mouse Anti-HSP90	Proteintech	60318-1-Ig	WB (1:1000)
Mouse Anti-GST	Sangon Biotech	D199985	WB (1:1000)
Rabbit Anti-SAPK/JNK	Cell Signaling	9252s	WB (1:1000)
Rabbit Anti-Phospho-SAPK/JNK	Cell Signaling	4668s	WB (1:1000)
Rabbit Anti-Thiophosphate ester	Abcam	ab92570	WB (1:1000)
Rabbit Anti-c-Myc Agarose Affinity Gel	Sigma	A7470	IP (1:100)
Mouse Anti-FLAG magnetic beads	Sigma	M8823	IP (1:200)
Mouse Anti-HA magnetic beads	Thermo Fisher	88837	IP (1:200)
